# Supplementary material for: Changes in Invasive Pneumococcal Disease Caused by Streptococcus pneumoniae Serotype 1 following Introduction of PCV10 and PCV13: Findings from the PSERENADE Project
Source: Microorganisms. 2021 Mar 27;9(4):696. doi: 10.3390/microorganisms9040696 (PMC8066231; doi:10.3390/microorganisms9040696)
Supplement: Supplementary file 1 [file microorganisms-09-00696-s001.pdf]

Changes in Invasive Pneumococcal Disease caused by *Streptococcus pneumoniae* Serotype 1 Following Introduction of PCV10 and PCV13: Findings from the PSERENADE project

Table of Contents

Supplementary Table 1. Sites included in PSERENADE evaluated for the serotype 1 analysis by age group 2

Supplementary Figure 1. Method for estimating annual ST1 IPD IRRs comparing the pre-PCV10/13 period to each post-PCV10/13 year for each included site with pre- and post-PCV10/13 data: example for children <5 years from one site. 5

Methods: Estimating annual ST1 IPD IRRs comparing the pre-PCV10/13 period to each post-PCV10/13 year using a Bayesian multi-level, mixed-effects Poisson regression 6

Supplementary Figure 2. All-site weighted average IRRs for ST1 IPD for all ages and by age group adjusted for pre-PCV trends in all-serotype IPD (n=30 sites) 7

Supplementary Figure 3. Site-specific modeled ST1 IPD IRRs by PCV product and age group. 8

Supplementary Figure 4. Site-specific modeled ST1 IPD IRRs by region and age group. 9

Supplementary Figure 5. Site-specific modeled ST1 IPD IRRs by infant PCV10/13 schedule and age group. 10

Supplementary Figure 6. Site-specific modeled ST1 IPD IRRs for adults ≥65 years by adult pneumococcal polysaccharide vaccine recommendation. 11

Supplementary Figure 7. Incidence rate of ST1 meningitis cases from cerebrospinal fluid (CSF) in Brazil by age group and year relative to PCV10 introduction (n=51 cases). 12

References 13

Supplementary Table 1. Sites included in PSERENADE evaluated for the serotype 1 analysis by age group (Y= Yes, included; N= No, excluded)

Site	Age Group				
	**<5 years**	**5-17 years**	**18-49 years**	**50-64 years**	**≥65 years**
**Australia (Non-Indigenous)**	Y	Y	Y	Y	Y
**Northern Territory, Australia**	Y	Y	Y	Y	Y
**Mirzapur, Bangladesh**	N[a]	--	--	--	--
**Belgium**	Y	Y	--	--	--
**Brazil**	N[d]	N[d]	N[d]	N[d]	N[d]
**Alberta, Canada**	Y	N[c]	Y	Y	Y
**Ontario, Canada**	N[c]	N[c]	Y	Y	Y
**Quebec (excluding Nunavik), Canada**	Y	Y	Y	Y	Y
**Quebec-Nunavik, Canada**	Y	N[b]	N[b]	N[b]	N[b]
**Metropolitan Region, Chile**	Y	Y	Y	Y	Y
**Non-Metropolitan Regions, Chile**	Y	Y	Y	Y	Y
**Czech Republic**	Y	Y	Y	Y	Y
**Denmark**	Y	Y	Y	Y	Y
**Fiji**	N[b]	N[a]	N[a]	N[a]	N[a]
**Finland**	Y	Y	Y	Y	Y
**France**	Y	Y	Y	Y	Y
**Basse, The Gambia**	Y	Y	N[a]	N[a]	N[a]
**Germany**	Y	Y	Y	Y	Y
**Greece**	N[d]	N[d]	N[d]	N[d]	N[d]

**Hong Kong**	N[c]	N[c]	Y	N[c]	N[c]
**Iceland**	Y	Y	Y	Y	Y
**Ireland**	Y	Y	Y	Y	Y
**Israel**	Y	Y	Y	Y	Y
**Italy**	Y	Y	Y	Y	Y
**Japan**	Y	Y	N[c]	N[c]	Y
**Asembo, Kenya**	Y	Y	Y	N[a]	N[a]
**Kibera, Kenya**	N[b]	N[b]	N[b]	N[a]	N[a]
**Kilifi, Kenya**	Y	Y	Y	Y	N[c]
**Latvia**	N[a]	N[a]	N[c]	Y	Y
**Blantyre District, Malawi**	Y	Y	Y	Y	Y
**Mongolia**	N[b]	--	--	--	--
**Morocco**	Y	Y	Y	--	N[a]
**Netherlands**	Y	Y	Y	Y	Y
**New Zealand**	Y	Y	Y	Y	Y
**Norway**	Y	Y	Y	Y	Y
**Poland**	Y	Y	N[a]	N[a]	N[a]
**Singapore**	N[c]	N[c]	N[c]	Y	Y
**Slovakia**	Y	Y	Y	Y	Y
**Slovenia**	Y	Y	Y	Y	Y
**South Africa**	Y	Y	Y	Y	Y
**Catalonia, Spain**	Y	Y	Y	Y	Y

**Madrid, Spain**	Y	Y	Y	Y	Y
**Navarra, Spain**	Y	Y	Y	Y	Y
**Sweden**	Y	Y	Y	Y	Y
**Switzerland**	Y	Y	Y	Y	Y
**England & Wales, UK**	Y	Y	Y	Y	Y
**Scotland, UK**	Y	Y	Y	Y	Y
**Active Bacterial Core surveillance (ABCs), USA**	Y	Y	Y	Y	Y
**Alaska, USA**	Y	Y	Y	Y	Y
**Massachusetts, USA**	Y	N[c]	--	--	--
**Southwest, USA (Indigenous)**	Y	Y	Y	Y	Y
**Utah, USA**	N[c]	--	--	--	--

[a] Biases in surveillance system over time that could not be accounted for
[b] Low proportion of cases serotyped
[c] Zero ST1 cases in all years
[d] Population-based surveillance data only available for pneumococcal meningitis cases
-- Data not provided or not available
Analyses were done with minor changes to age groups for certain sites to align with availability of population denominators and age groups provided by sites in aggregate: the <5 years age group includes 0-5 years from Morocco; the 5-17 years age group included 5-14 years from Japan and Kilifi, Kenya, 5-15 years from Germany, 6-14 years from Morocco, and 5-19 years from Australia and Malawi; and the 18-49 years age group includes 15-49 years from Japan and Kilifi, Kenya, 15-59 years from Morocco, 16-49 years from Germany, and 20-49 years from Australia and Malawi.

Supplementary Figure 1. Method for estimating annual ST1 IPD IRRs comparing the pre-PCV10/13 period to each post-PCV10/13 year for each included site with pre- and post-PCV10/13 data: example for children <5 years from one site.

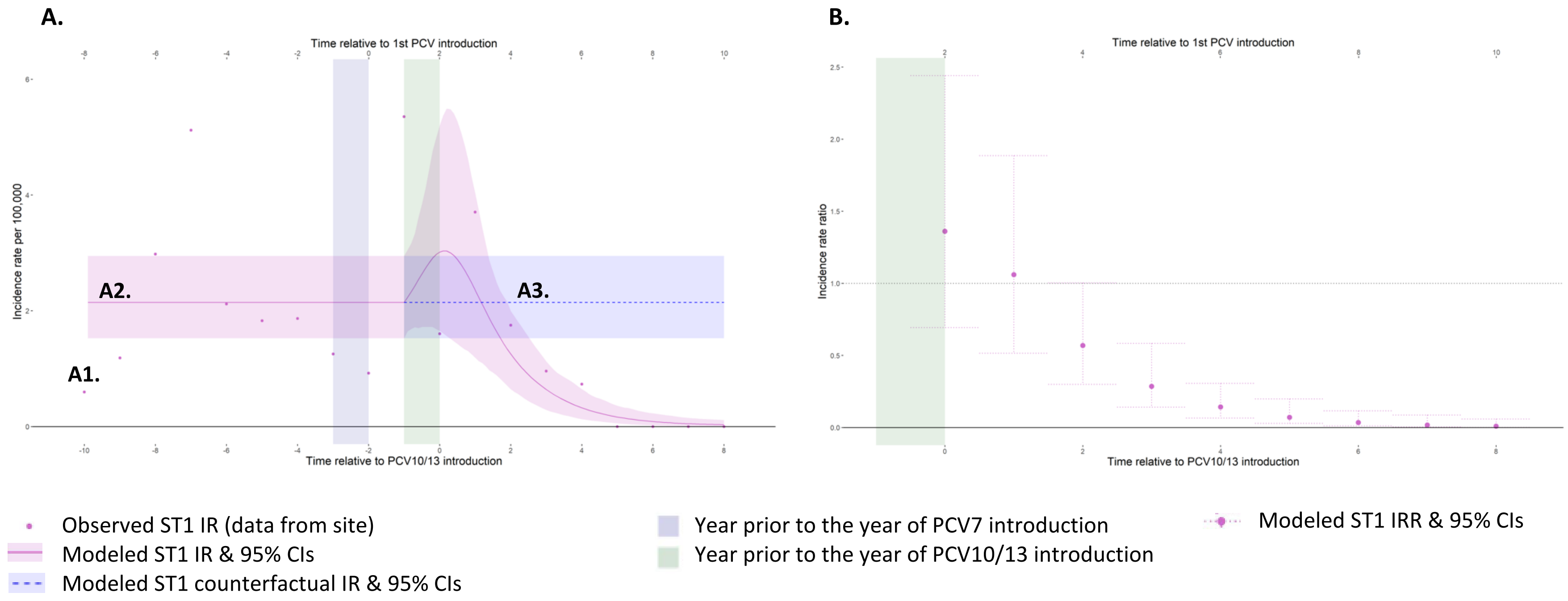

A. Annual ST1 IRs.

A1. Observed ST1 IR (data received from site).

A2. Modeled site-specific IR and surrounding 95% CIs estimated for years of available data using a Bayesian multi-level, mixed-effects Poisson regression including data from all other sites for children <5 years with an offset for population denominator and random effects for the intercept and slope. A non-linear break is included one year prior to PCV10/13 introduction to capture the change from the pre-PCV10/13 period to the year of PCV10/13 introduction and cubic spline knots are included at year +1 and +3 post-PCV10/13 introduction to allow for flexibility in the IR of ST1 following PCV10/13 introduction.

A3. Counterfactual ST1 IR and surrounding 95% CIs in the absence of PCV10/13 introduction.

B. Annual modeled ST1 IRRs in each post-PCV10/13 year. IRRs are estimated by dividing the modeled ST1 IR in each post-PCV10/13 year by the counterfactual IR.

Methods: Estimating annual ST1 IPD IRRs comparing the pre-PCV10/13 period to each post-PCV10/13 year using a Bayesian multi-level, mixed-effects Poisson regression

A Bayesian multi-level, mixed-effects Poisson regression was used to estimate annual ST1 IPD IRs across all years of available data for each site using the MCMCglmm package in R [1]. The model included an offset for population denominator and random effects for all of the site-specific regression coefficients that allows for heterogeneity among sites in the shapes of their infection curves. A site-specific non-linear break (a pre-/post-PCV10/13 indicator variable that interacted with the linear function of time) was included in the model one year prior to PCV10/13 introduction to capture the change from the pre-PCV10/13 period to the year of PCV10/13 introduction. Cubic splines knots were included for each site at years +1 and +3 (second and fourth year of PCV10/13 use) to allow for flexibility in the IR of ST1 over time for each site following PCV10/13 introduction. Cubic spline knots were concentrated in the early part of the post-PCV10/13 period where IR changes were expected to be the most dramatic and heterogenous across sites. We used default conjugate priors for the regression coefficients and for the elements in the variance comatrix for the random effects. We ran the model with 20,000 MCMC iterations, a burnin of 10,000 and a thinning interval of 40, resulting in 250 samples. We modeled the pre-PCV10/13 ST1 IRs as a flat line (slope of zero) and extended these rates into the post-PCV10/13 period as the counterfactual ST1 IRs. The site-specific modeled ST1 IR and counterfactual IR were used to estimate site-specific annual IRRs in each post-PCV10/13 year with the mean of the posterior distribution of rate ratios (the modeled ST1 IR in each post-PCV10/13 year: the counterfactual ST1 IR) for each site. CIs were estimated using the 2.5 and 97.5 percentiles of the posterior distribution of the IRs.

Supplementary Figure 2. All-site weighted average IRRs for ST1 IPD for all ages and by age group adjusted for pre-PCV trends in all-serotype IPD (n=30 sites)

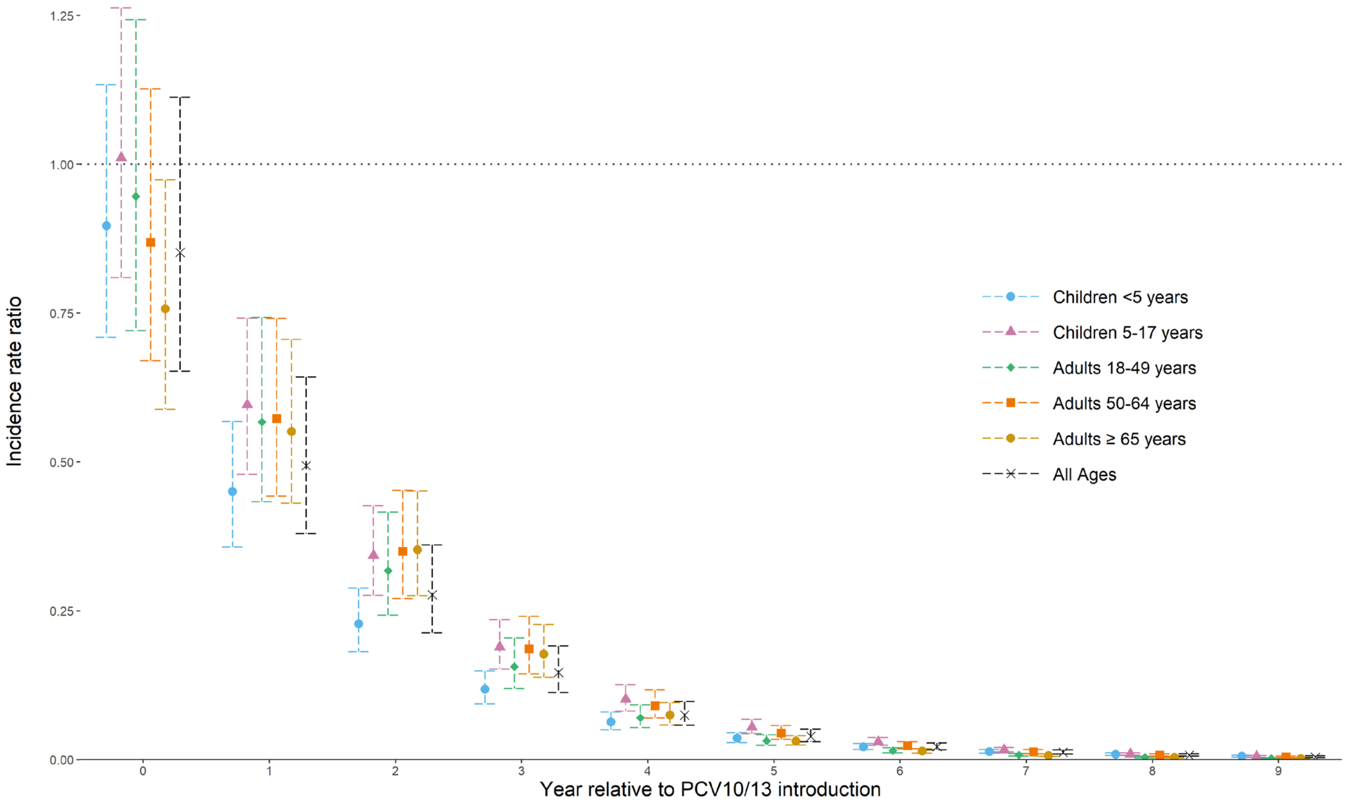

To account for potential changes in IPD surveillance sensitivity or general health improvements prior to the introduction of PCV (including PCV7, if used), we performed a sensitivity analysis adjusting the pre-PCV ST1 case counts by trends in all-serotype IPD prior to PCV introduction for sites with both pre- and post-PCV data.

Supplementary Figure 3. Site-specific modeled ST1 IPD IRRs by PCV product and age group.

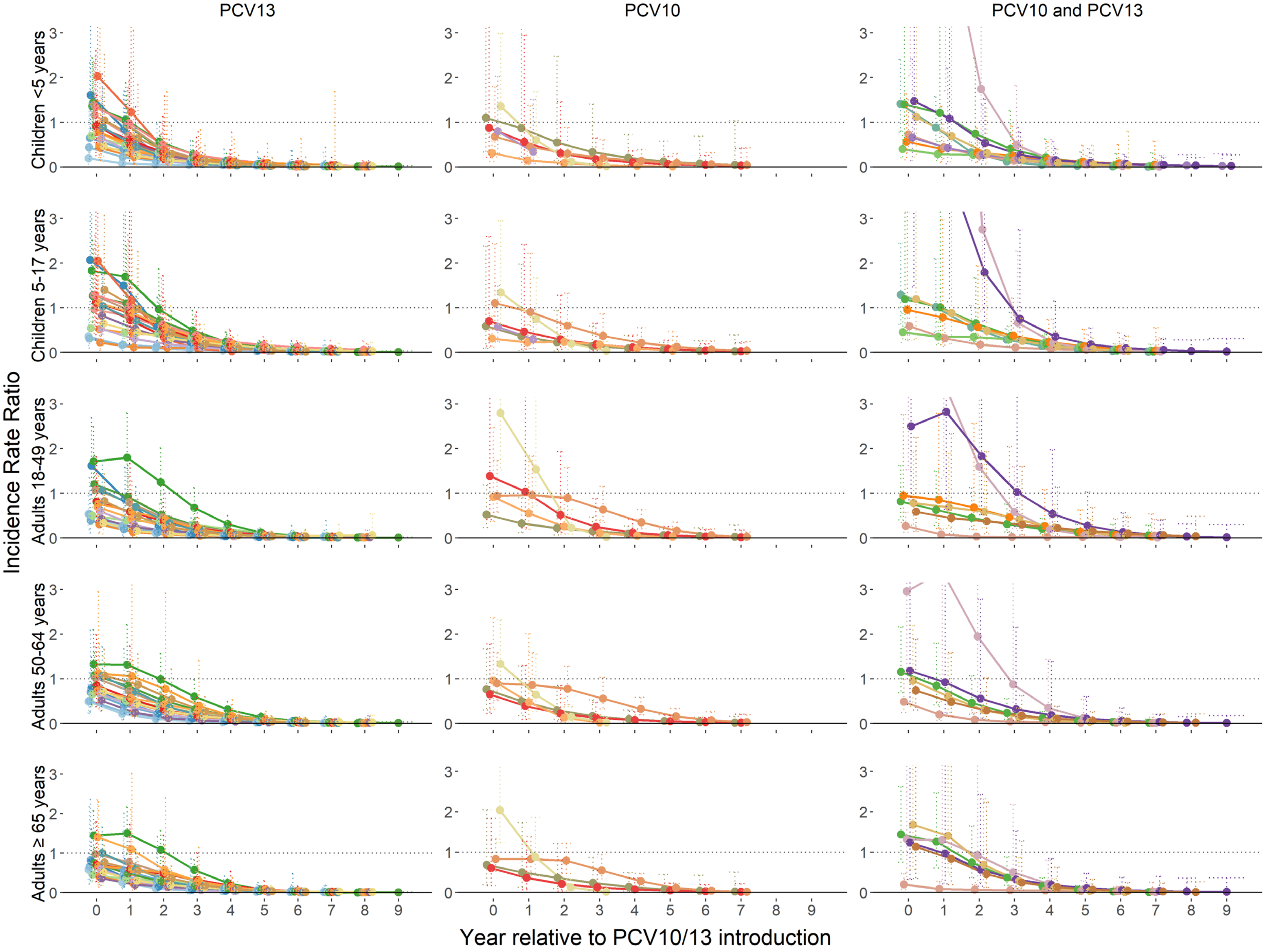

'PCV10 and PCV13' sites either switched between products or used both products concurrently.

Supplementary Figure 4. Site-specific modeled ST1 IPD IRRs by region and age group.

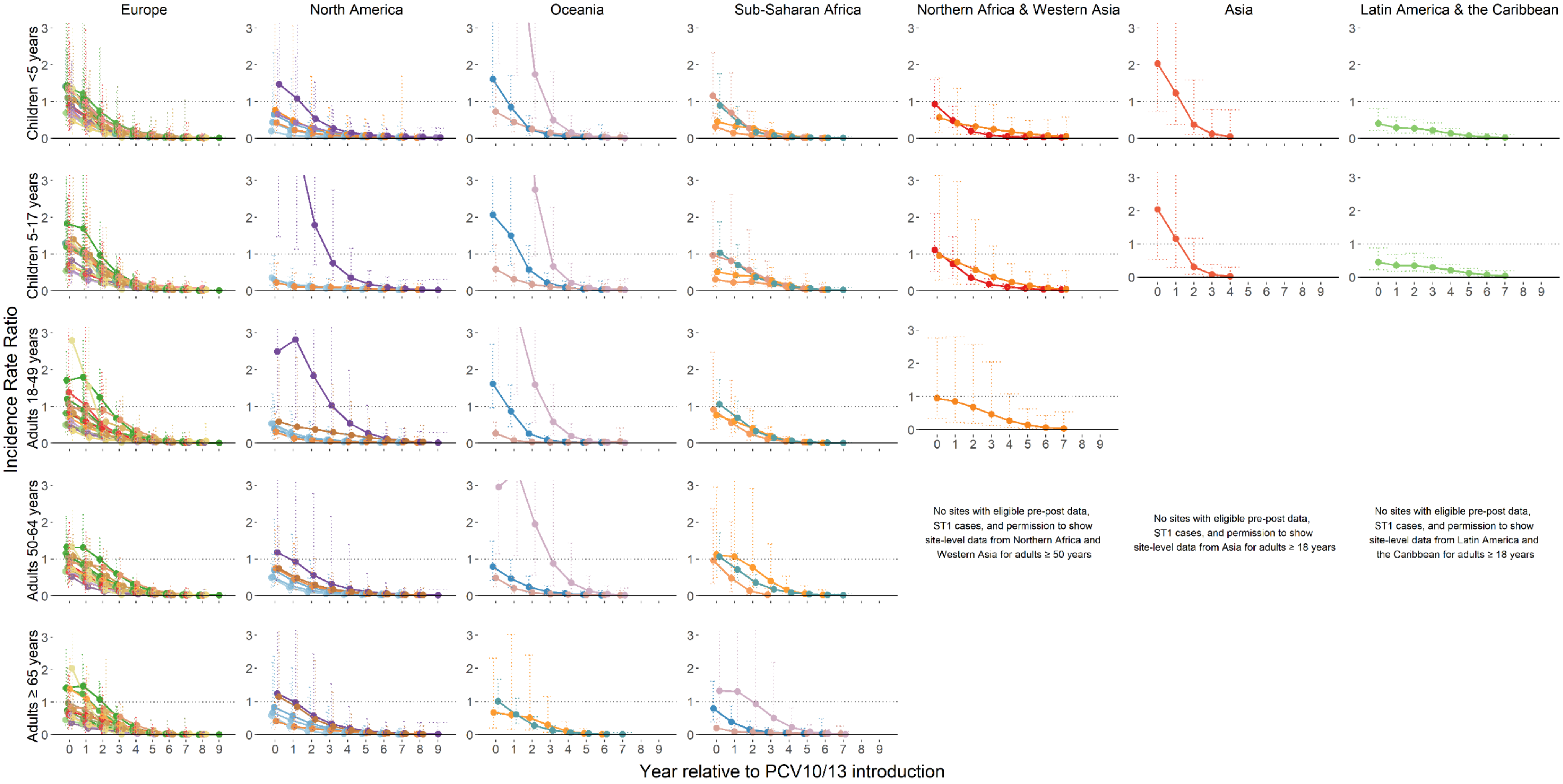

Supplementary Figure 5. Site-specific modeled ST1 IPD IRRs by infant PCV10/13 schedule and age group.

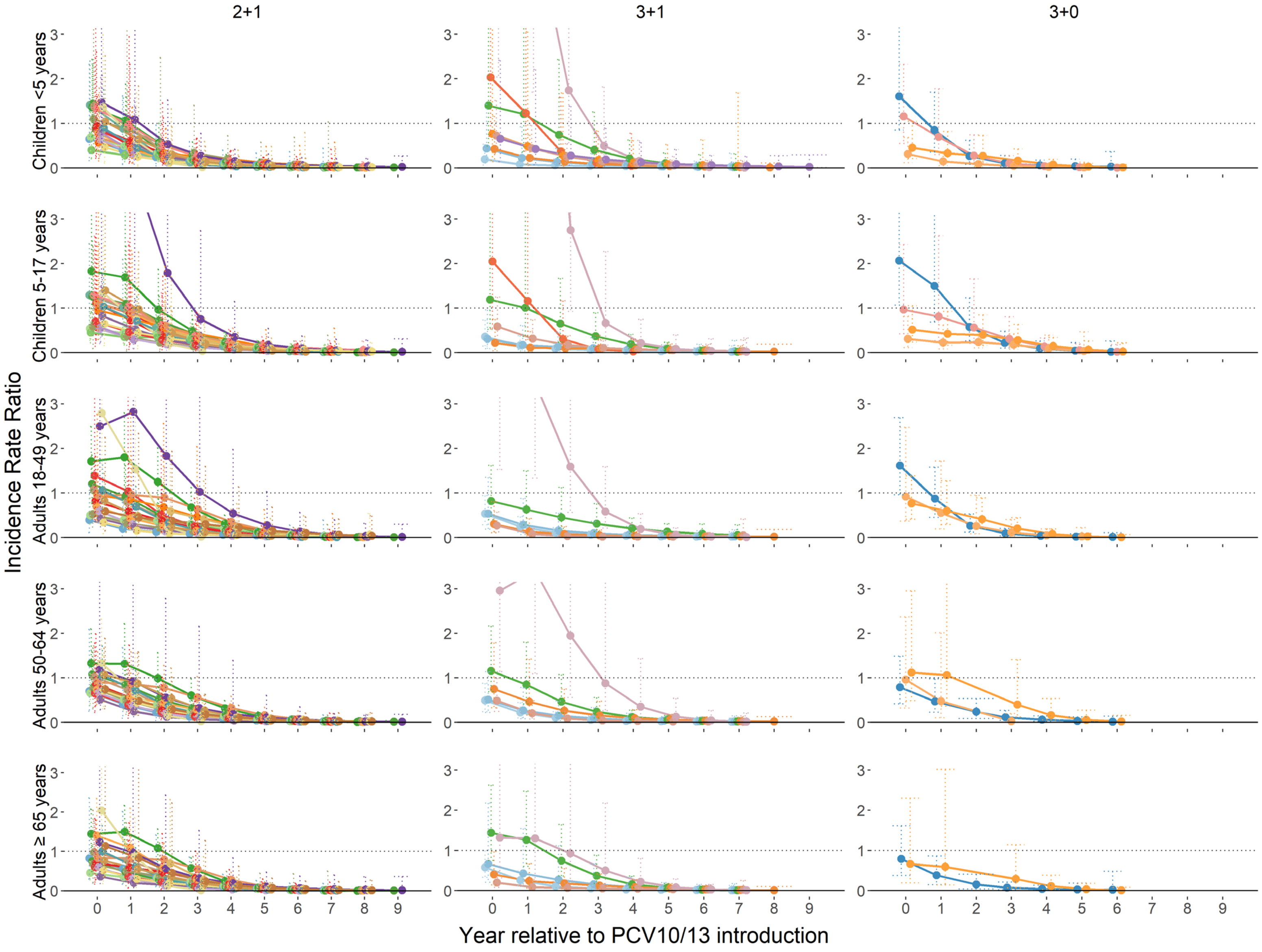

Supplementary Figure 6. Site-specific modeled ST1 IPD IRRs for adults ≥65 years by adult pneumococcal polysaccharide vaccine recommendation.

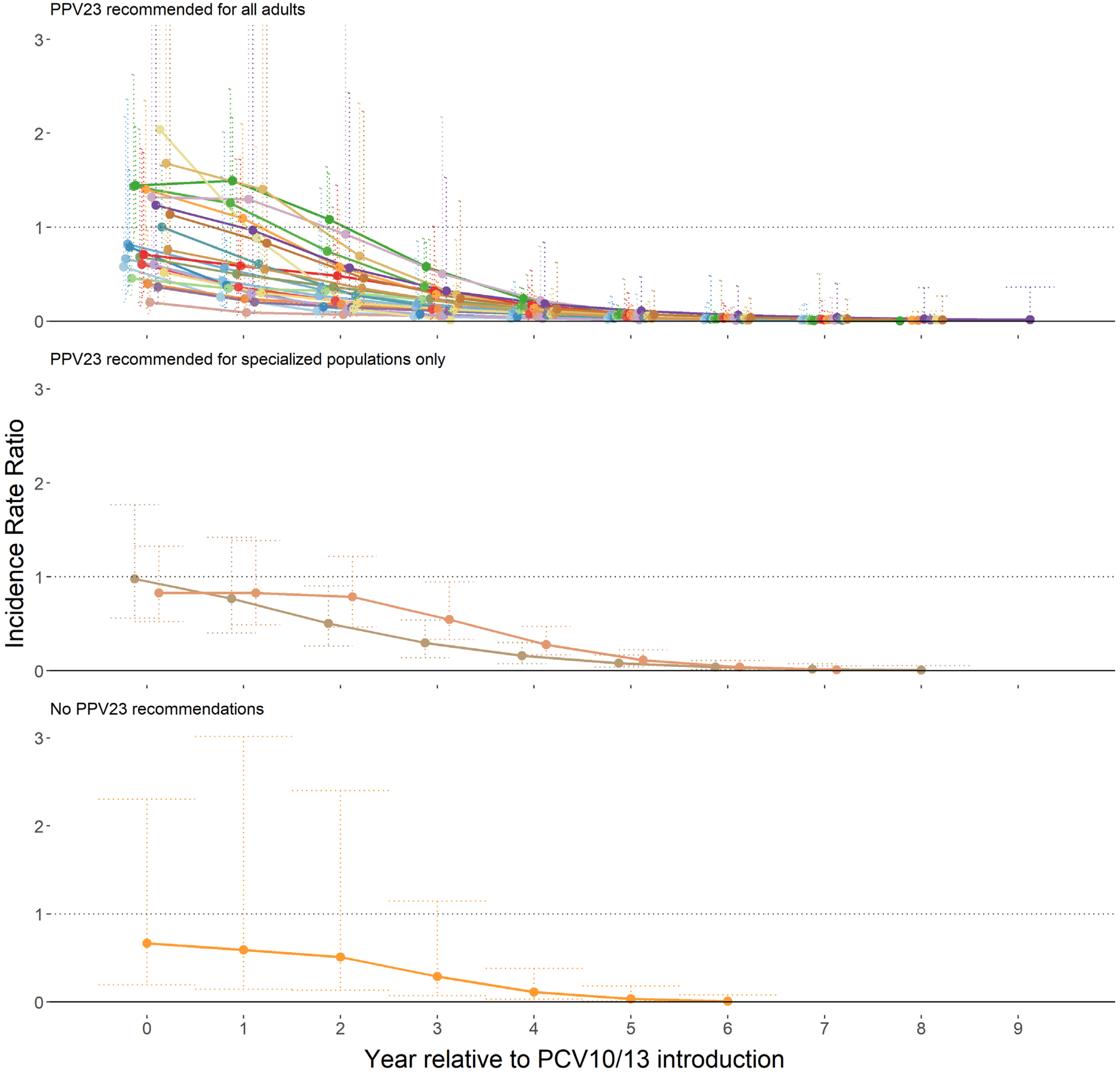

Supplementary Figure 7. Incidence rate of ST1 meningitis cases from cerebrospinal fluid (CSF) in Brazil by age group and year relative to PCV10 introduction (n=51 cases).

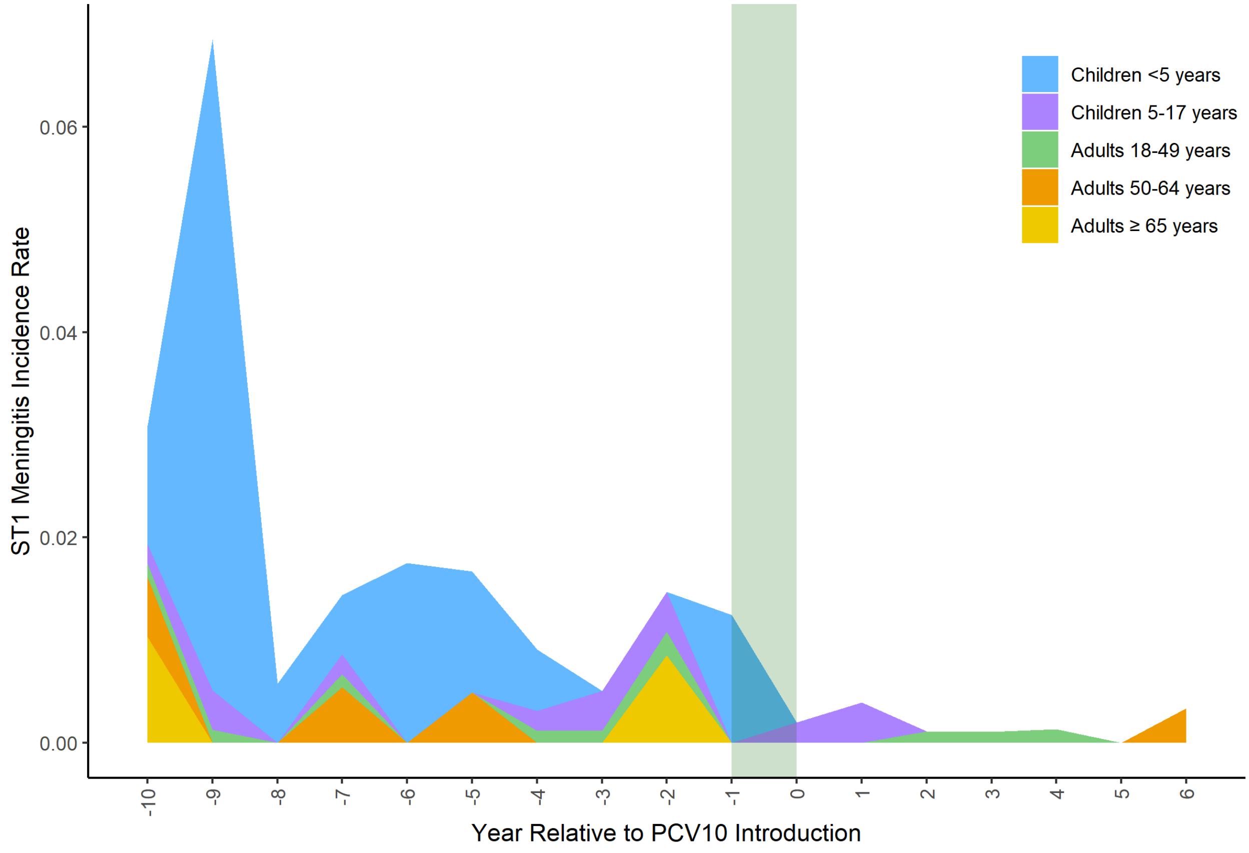

Green bar shades the change from the year prior to the year of PCV10 introduction.

References

1. Hadfield, J.D. MCMC Methods for Multi-Response Generalized Linear Mixed Models: The MCMCglmm R Package. *J. Stat. Softw.* **2010**, *33*, 1–22, doi:10.18637/jss.v033.i02.
